# Non-tuberculous Mycobacteria in South African Wildlife: Neglected Pathogens and Potential Impediments for Bovine Tuberculosis Diagnosis

**DOI:** 10.3389/fcimb.2017.00015

**Published:** 2017-01-30

**Authors:** Nomakorinte Gcebe, Tiny M. Hlokwe

**Affiliations:** Tuberculosis Laboratory, Onderstepoort Veterinary Institute, Zoonotic Diseases, Agricultural Research CouncilOnderstepoort, South Africa

**Keywords:** non-tuberculous mycobacteria, South African wildlife, bovine tuberculosis, 16S rDNA sequencing, immune responses, potential pathogens

## Abstract

Non-tuberculous mycobacteria (NTM) are not only emerging and opportunistic pathogens of both humans and animals, but from a veterinary point of view some species induce cross-reactive immune responses that hamper the diagnosis of bovine tuberculosis (bTB) in both livestock and wildlife. Little information is available about NTM species circulating in wildlife species of South Africa. In this study, we determined the diversity of NTM isolated from wildlife species from South Africa as well as Botswana. Thirty known NTM species and subspecies, as well as unidentified NTM, and NTM closely related to *Mycobacterium goodii/Mycobacterium smegmatis* were identified from 102 isolates cultured between the years 1998 and 2010, using a combination of molecular assays viz PCR and sequencing of different Mycobacterial house-keeping genes as well as single nucleotide polymorphism (SNP) analysis. The NTM identified in this study include the following species which were isolated from tissue with tuberculosis- like lesions in the absence of *Mycobacterium tuberculosis* complex (MTBC) implying their potential role as pathogens of animals: *Mycobacterium abscessus* subsp. *bolletii, Mycobacterium gastri, Mycobacterium* species closely related to *Mycobacterium goodii/Mycobacterium smegmatis, Mycobacterium brasiliensis, Mycobacterium sinense* JMD 601, *Mycobacterium avium* subsp. *avium, Mycobacterium* sp. GR-2007, *Mycobacterium bouchedurhonense*, and *Mycobacterium septicum*/*M. peregrinum. Mycobaterium brasiliensis, Mycobacterium gastri, Mycobacterium* sp. GR-2007, and a potential novel *Mycobacterium* species closely related to *Mycobacterium goodii* were found for the first time in this study to be potential pathogens of animals. *Mycobacterium simiae* was isolated from a sample originating from a tuberculin skin test positive reactor, demonstrating its potential to elicit inappropriate immune responses in animals that may interfere with diagnosis of tuberculosis by immunology. *Mycobacterium abscessus* subsp. *bolletti* was the most frequently detected NTM identified in 37 of the 102 isolates. Other NTM species were also isolated from animals not showing any pathological changes. Knowledge gained in this study contribute to the understanding of NTM species circulating in wild animals in South Africa and the pathogenic potential of certain species, whose role in disease causation need to be examined, as well as to a certain extent the potential of *M. simiae* to hamper the diagnosis of bTB.

## Introduction

Non-tuberculous mycobacteria (NTM), otherwise known as “mycobacteria other than tuberculosis” (MOTT) or environmental mycobacteria (EM) are believed to be natural inhabitants of the environment, found as saprophytes, commensals, and symbionts in the ecosystem. Since their clinical relevance was unknown, these bacteria have been neglected for many years as they have always been recognized as just environmental contaminants or colonizers (Covert et al., 1999). It was only after the acquired immune deficiency syndrome (AIDS) epidemic that NTM attracted abrupt attention in certain countries (Gopinath and Singh, 2010). Finding immune compromised hosts, especially with the emergence of the AIDS pandemic, some NTM species are now recognized as potential opportunistic pathogens of humans (Mirsaeidi et al., 2014). However, other than a few known NTM pathogens of animals, including the well-known *Mycobacterium avium* subsp. *paratuberculosis*, the etiological agent of Johne's disease and other members of the *Mycobacterium avium* complex (MAC), reports of animal diseases caused by NTM are few.

Currently there are more than 150 non-tuberculous mycobacterial species listed in public databases and about a third of them have been implicated in diseases of humans. (http://www.bacterio.net; Botha et al., 2013). Because of the ubiquity of NTM, human infections have been reported from most geographic areas in the world and the geographical distribution is thought to be regional (Martin-Casabona et al., 2004; van Ingen et al., 2009). Mycobacterial diseases caused by NTM have been classified as skin lesions, localized lymphadenitis, TB-like pulmonary lesions and disseminated diseases (Primm et al., 2004). By far the most notable opportunistic NTM pathogens of animals and humans are the members of the *Mycobacterium avium* complex (MAC) consisting of closely related species and subspecies including *Mycobacterium avium* subsp. *avium, Mycobacterium avium* subsp. *paratuberculosis, Mycobacterium avium* subsp. *hominissuis, Mycobacterium intracellulare, Mycobacterium sylvaticum, Mycobacterium colombiense, Mycobacterium bouchedurhonense, Mycobacterium timonense, Mycobacterium chimaera, Mycobacterium arosiense, Mycobacterium yongonense*, and *Mycobacterium marseillense* (Cayrou et al., 2010). Other common NTM species reported to cause opportunistic mycobacterial diseases include *Mycobacterium kansasii, Mycobacterium marinum*, and *Mycobacterium ulcerans*. Animal infections by *M. kansasii* are very rare (Botha et al., 2013). Infections by *Mycobacterium haemophilum, Mycobacterium szulgai, Mycobacterium fortuitum, Mycobacterium chelonae, Mycobacterium wolinskyi, Mycobacterium scrofulaceum, Mycobacterium abscessus, Mycobacterium xenopi, Mycobacterium genavense, Mycobacterium fortuitum, Mycobacterium porcicum, Mycobacterium farcinogens, Mycobacterium senegalense*, and *Mycobacterium genavense* have also been reported in humans or animals. These NTM species have been reported to be etiology of mycobacterial diseases in humans and a wide range of animal species including livestock, wildlife amphibians, fish, reptiles, seals, birds, and rodents (Tortoli et al., 1998; Bercovier and Vincent, 2001; Martin-Casabona et al., 2004; van Ingen et al., 2008; Malama et al., 2014).

There are even fewer reports of NTM isolated from wildlife in Africa. Studies on isolation of NTM from animal sources have mainly focused on either livestock from slaughter houses or on NTM that were coincidentally isolated from animal tissue, including livestock and wildlife, while looking for *Mycobacterium bovis* (Kazwala et al., 1998; Tschopp et al., 2010). The extent of NTM infection and distribution in African wildlife is therefore largely unknown. In a study in Ethiopian cattle, *Mycobacterium nonchromogenicum* was isolated as a predominant species (Berg et al., 2009) while *Mycobacterium terrae* was isolated as a frequently occurring species in Ethiopian wildlife (Tschopp et al., 2010). A study from Chad found *M. nonchromogenicum* together with MAC and *M. fortuitum* to be common in humans and cattle (Diguimbaye-Djaïbe et al., 2006). *Mycobacterium terrae* was isolated from cattle in Tanzania (Kazwala et al., 1998; Cleaveland et al., 2007). *M. nonchromogenicum* has also been detected in small mammals and cattle in Tanzania (Durnez et al., 2011). A recent study in Tanzania identified *Mycobacterium intracellulare, Mycobacterium lentiflavum, Mycobacterium fortuitum*, and *Mycobacterium chelonae-abscessus* group as the most frequently isolated NTM from humans and wildlife (Katale et al., 2014). It should be noted that, isolation of NTM from an animal source does not necessarily imply an active disease status. *Mycobacterium goodii* was reported to have caused infection in a hyena in South Africa as well as in African rodents in Tanzania and *M. kansasii* has been isolated from wild animal (Bercovier and Vincent, 2001; van Helden et al., 2009; Durnez et al., 2011). *M. farcinogenes* and *M. senegalense* were reported to be responsible for bovine farcy, pathology found in Africa (Bercovier and Vincent, 2001). The environment is also of interest as the source of NTM infections (van Ingen et al., 2009). In Uganda, *M. nonchromogenicum, M. fortuitum* complex, *M. avium* complex, and *M. gordonae* were identified as most frequently detected species from environmental samples (Kankya et al., 2011). A study in Zaire also identified *M. nonchromogenicum* and *M. terrae* to be among the most prevalent NTM species in the environment (Portaels, 1995). In South Africa the following NTM have been identified as most occurring in different studies: Gcebe et al. (2013) have found the following NTM to be frequently occurring in African buffaloes, cattle and their environments: *Mycobacterium terrae, M. nonchromogenicum, M. vaccae/M. vanbaalenii*, and unidentified species closely related to *Mycobacterium moriokense*. They also noted an overlap in terms of the species isolated from the animal sources as well as the environments indicating that NTM are readily exchanged between the environment and animals (Gcebe et al., 2013). Botha et al. (2013) have reported isolation of the following NTM from different wildlife species including lions, rhinos, banded mongooses, cattle, baboons, elephants, and monkeys: *M. abcessus, M. asiaticum, M. avium, M. brasilienses, M. chelonae, M. elephantis, M. engbackii, M. farcinogenes, M. fortuitum, M. gilven, M. gordonae, M. heraklionense, M. hiberniae, M. intracellulare, M. interjectum, M. lentiflavum, M. marseillense, M. moriokaense, M. nonchromogenicum, M. palustre, M. pulveris, M. paraffinicum, M. phlei, M. senegalense, M. simiae, M. sherrisii, M. sphagni, M. terrae*, and *M. vulneris. Mycobacterium gordonae* and *Mycobacterium gilvum* were identified as the most frequently occurring NTM in biofilms, in a study by September et al. (2004).

In Africa, high interaction between wild animals especially captive and semi-captive, the environment, and humans seem to be very common posing a risk of NTM contamination of natural waters and transmission of NTM between animals and humans (Katale et al., 2014).

From a veterinary point of view, besides being opportunistic pathogens, some NTM species may colonize the host without development of any disease, but inappropriately prime the host's immune system hampering the immuno-diagnosis of bovine tuberculosis by tuberculin based assays (Vordermeier et al., 2007; Schiller et al., 2010). Furthermore, NTM may have a negative effect on vaccination of animals by *Mycobacterium bovis* Bacillus Calmette-Guérin (BCG; Poyntz et al., 2014). Incidents of bovine tuberculosis in different South African wildlife have been reported, and efforts for eradication or reduction of the diseases to minimal levels include the test and slaughter strategies (Hlokwe et al., 2014). Vaccination is also a possibility. Yet very little information is available on NTM circulating in wild animals in South Africa. Since, bovine tuberculosis (bTB) is endemic in South African wildlife conservation areas, specifically, in the Kruger National Park, incidences of NTM infection of wildlife may have been neglected probably due to the fact that focus has been on bTB, and NTM infection may be last on the agenda or perceived to be less important. The irony is that the interference of NTM in the diagnosis of bTB as well is in vaccination by BCG may hinder the bTB control/eradication efforts in wildlife of South Africa.

In this study, we determined the diversity of NTM species isolated from different wildlife species of South Africa and Botswana.

This study forms the basis for understanding the diversity of NTM species which are circulating in the South African wildlife as well as potential pathogens of different animal species as well as NTM that may have potentially induced in-appropriate immune responses which interfered with the diagnosis of tuberculosis. This information may hopefully aid in the efforts for control of bovine tuberculosis in wildlife through development of diagnostic assays and vaccination strategies.

## Materials and methods

### Ethics statement

This work was carried out from samples submitted for routine diagnostic purposes in the Tuberculosis laboratory: a Veterinary Laboratory of South Africa approved by the Department of Agriculture, Forestry, and Fisheries (DAFF) in compliance with the requirements of the Animal Diseases act no. 35 of 1984 as well as DAFF 001, 002, and 012 procedures.

### Study site

Isolates originating from samples collected for bovine tuberculosis or mycobacterial routine diagnosis from the Greater Kruger National Park (GKNP) complex, the National Zoological Gardens (NZG) of South Africa, and four private game reserves, were used in this study. The Greater Kruger National Park complex consists of several game parks/reserves and covers the area in the north east of South Africa, covering 19.485 km^2^. National Zoological Gardens of South Africa is located in Pretoria, north of the Gauteng Province north east of South Africa, covering 0.85 km^2^. Three of the private game reserves are located in the Mpumalanga Province, east of South Africa, and one is located in the Northern Cape Province, north west of South Africa. Isolates from mongoose and wildcat from Botswana were also included in the study.

### Sample collection from wildlife

Samples were collected opportunistically from dead as well as live animals. Dead animals included road kills, animals that died from disease or old age and animals that were culled for bTB confirmation after they tested positive on tuberculin skin test (TST). One hundred and two (102) tissue samples including bronchial, mesenteric, thoracic, peripheral, abdominal, mammary, axial, popliteal, cranial, and head lymph nodes as well as lungs, liver, testicles, heart, tonsils, bone marrow, ulna, nose, bronchial washes, and elbow hygroma were collected between the years 1998 and 2010. Information regarding the different animal species sampled and their geographic origin is presented in Table 1.

**Table 1:** **Different wildlife species sampled and their geographic origin**.

**Animal species**	**Total number of animals sampled**	**Geographic origin (*n* = number of samples)**
Lion	(*n* = 66)	GKNP (*n* = 60)
		Private game reserves in SA (*n* = 6)
Leopard	(*n* = 6)	GKNP in SA (*n* = 4)
		NZG in SA (*n* = 2)
Hyena	(*n* = 4)	GKNP in SA (*n* = 4)
White rhino	(*n* = 2)	Private game reserves in SA (*n* = 2)
Eland	(*n* = 2)	Private game reserves in SA (*n* = 2)
Vervet monkey	(*n* = 2)	Private game reserves in SA (*n* = 2)
Black wildebeest	(*n* = 2)	Private game reserves in SA (*n* = 2)
Cheetah	(*n* = 1)	GKNP in SA (*n* = 1)
Kudu	(*n* = 1)	GKNP in SA (*n* = 1)
Spotted genet	(*n* = 1)	Private game reserve in SA (*n* = 1)
Meerkat	(*n* = 1)	Private game reserve in SA (*n* = 1)
Orang-utan	(*n* = 1)	NZG in SA (*n* = 1)
Lichtenstein hartebeest	(*n* = 1)	GKNP in SA (*n* = 1)
Wild cat	(*n* = 1)	Botswana (*n* = 1)
Mongoose	(*n* = 7)	Botswana (*n* = 7)
Nyala	(*n* = 1)	GKNP in SA (*n* = 1)
Bushbuck	(*n* = 1)	GKNP in SA (*n* = 1)
African rodent	(*n* = 1)	NZG in SA (*n* = 1)
Blesbok	(*n* = 1)	GKNP in SA (*n* = 1)

### Pathological examination and culture

Tissue samples to be processed for mycobacterial isolation were carefully examined macroscopically for formation of suspected tuberculous-like lesions (visible). The presence or absence of suspect bTB-like lesion was recorded for each sample.

All tissue samples were processed and cultured according to standard laboratory procedures in a Class II biosafety cabinet (Esco Technologies, South Africa) following biosafety guidelines as described in the OIE Terrestrial manual (OIE, 2015). Briefly, ~5 g of tissue samples were cut into small pieces and covered with 100 ml of sterile distilled water. The samples were homogenized at 4500 rpm using the Ultra-Turrax® homogenizer (Separation Scientific, SA). Seven milliliters of the homogenates were poured into two separate 15 ml falcon tubes, and decontaminated with 7 ml of 2% HCl and 7 ml of 4% NaOH, respectively. The suspensions were incubated at room temperature for 10 min and centrifuged again. Following centrifugation, sterile distilled water was added to the pellet. The pellet was inoculated onto four Löwenstein-Jensen (LJ) media slopes supplemented with pyruvate, as well as two slopes supplemented with glycerol and an antibiotic cocktail of polymyxin B, ampho-tericin B, carbenicillin, and trimethoprim (PACT) (National Health Laboratories, South Africa, and Becton Dickinson, South Africa). The slopes were incubated at 37°C and monitored weekly for mycobacterial growth. When growth of bacteria was observed, based on morphology of mycobacterial colonies, individual colonies were selected for Ziehl–Neelsen staining. The acid fast cultures were stored at −21°C for further analysis.

### DNA extraction

DNA was extracted using the heating method. Individual acid fast colonies were suspended in 100 μl of sterile distilled water and heated at 95°C in a heating block/or in boiling water for 25 min. The culture suspensions were stored at −70 or −20°C until further analysis.

### *In vitro* amplification and sequencing of the mycobacterial 16S rDNA and *hsp65* genes for NTM speciation

PCR and sequencing of the 577 bp fragment of the mycobacterial 16S rDNA was used for mycobacterial speciation (Harmsen et al., 2003; Gcebe et al., 2013). Sequencing of the 439 bp *hsp65* gene fragment was done for differentiation of some NTM species with similar, or identical 16S rRNA which could not be delineated using the 16S rDNA PCR-sequencing assay (Telenti et al., 1993).

#### 16S rDNA PCR

PCR targeting a 577 bp fragment of mycobacterial 16S rDNA was performed using the primers: 16S-F (5′-AGA GTT TGA TCM TGG CTCAG-3′) and 16S-R (5′-GCG ACA AAC CAC CTA AGA G-3′). Culture suspensions were used as DNA template in a 25 μl PCR mixture containing 12.9 μl deionized water, 2.5 μl of 10X PCR buffer [160 mM; Tris Cl, KCl, (NH_4_)_2_SO_4_], 2 μl MgCl_2_ (25 mM), 1 μl dNTPs (10 mM), 0.1 μl Taq polymerase (Qiagen Hotstar Taq, Whitehead Scientific, South Africa), 5 μl of 5x Q-solution, 1 μl of each forward and reverse primers (50 pmol) and 1–2 μl DNA template. The PCR cycling parameters were as follows: initial denaturation at 95°C for 15 min, followed by 35 cycles of denaturation at 95°C for 30 s, annealing at 60°C for 30 s, and elongation at 72°C for 30 s and a final extension at 72°C for 10 min.

#### *hsp65* PCR

Tb11 (5′-ACCAACGATGGTGTGTCCAT-3′) and Tb12 (5′-CTTGTCGAACCGCATACCCT-3′) primers were used for the amplification of the *hsp65* gene fragment. A 50 μl PCR mixture containing 28.5 μl de-ionized water, 3 μl MgCl_2_ (25 mM), 1 μl dNTP mix (10 mM), 4.75 μl of 10x PCR buffer (160 mM) [Tris-HCl, MgCl_2_, Tween 20, (NH_4_)_2_,SO_4_], 0.75 μl Taq DNA Polymerase (5 U/μl; Supertherm™), 1 μl of each forward and reverse primers (50 pmol) and 10 μl of DNA template was assembled. The PCR cycling parameters were as follows: Forty-five cycles of denaturation at 94°C for 1 min, annealing at 60°C for 1 min, and elongation at 72°C for 1 min and final extension at 72°C for 10 min.

The PCR products were sent to Inqaba Biotechnologies, South Africa for sequencing of the forward 16S rDNA strands and both *hsp65* gene strands using an ABI sequencer. Sequences were edited manually and pairwise alignments undertaken using the BioEdit Sequence alignment editor (version 7.1.9) and Molecular Evolutionary Genetics Analysis (MEGA) platform (www.megasoftware.net; version 7; Kumar et al., submitted). The sequences were analyzed on the NCBI BLAST platform for species identification (https://blast.ncbi.nlm.nih.gov/Blast.cgi) by megablast.

### PCR for detection of *Mycobacterium avium* subsp. *avium* and *Mycobacterium avium* subsp. *paratuberculosis*

To delineate between two members of MAC: i.e., *M. avium* subspecies *paratuberculosis* and *M. avium* subsp. *avium* which could not be differentiated by the genus specific 16S rDNA-sequencing assay, the MYCGEN-MYCAV PCR as described by Wilton and Cousins (1992) as well as amplification of the *IS900* of the *M. avium* subsp. *paratuberculosis* (Cousins et al., 1999) was conducted in two separate PCR reactions using primers described below:

MYCGEN-F (5′-AGAGTTTGATCCTGGCTCAG-3′), MYCGEN-R (5′-TGCACACAGGCCACAAGGGA-3′) and MYCAV (5′-ACCAGAAGACATGCGTCTTG- 3′) primers were used for screening of the *Mycobacterium avium* subspecies *avium* isolates as described by Wilton and Cousins (1992). Briefly a 25 μl PCR mixture was prepared, containing 14 μl de-ionized water, 2 μl MgCl_2_ (25 mM), 1 μl dNTP mix (10 mM), 1 μl of 10x PCR buffer [160 mM; Tris-HCl, MgCl_2_, Tween 20, (NH_4_)_2_, SO_4_], 0.125 μl Taq DNA Polymerase (5 U/μl; Supertherm™), 0.5 μl of each primer (50 pmol), and 5 μl of DNA template. The PCR cycling parameters were as follows: Initial denaturation at 94°C for 5 min, followed by 40 cycles of denaturation at 94°C for 30 s, annealing at 62°C for 3 min, and elongation at 75°C for 3 min.
IS*900* (P90) forward: 5′-GAAGGGTGTTCGGGGCCGTC-3′ and IS*900* (P91) reverse: 5′-GAGGTCGATCGCCCACGTGAC-3′ primers were used for screening of the isolates for *M. avium* subsp. *paratuberculosis* in 50 μl PCR reaction mixture containing 28 μl de-ionized water, 3 μl MgCl_2_ (25 mM), 2 μl dNTP mix (10 mM), 5 μl of 10x PCR buffer [160 mM; Tris -HCl, MgCl_2_, Tween 20, (NH_4_)_2_,SO_4_], 0.125 μl Taq DNA Polymerase (5 U/μl; Hotstar), 1 μl of each primer (50 pmol), and 10 μl of DNA template. The PCR cycling parameters were as follows: Initial denaturation at 95°C for 10 min, followed by fifty cycles of denaturation at 95°C for 1 min, annealing at 65°C for 75 s, and elongation at 72°C for 3 min. This was followed by a final elongation at 72°C for 10 min. Amplicons were analyzed by electrophoresis on a 1.5% agarose gel.

### Alignment of the 16S rDNA sequences and phylogenetic analysis

Multiple alignment of the part of the 16S rDNA sequences from representative isolate of each species identified in the study as well other NTM sequences (both slow and rapidly growing) extracted from NCBI Nucleotide database (https://www.ncbi.nlm.nih.gov/nuccore) was performed using MEGA version 7.0 platform (Kumar et al., submitted). For phylogenetic analysis the 16S rDNA sequences were first trimmed at both the 5′ and the 3′ ends to encompass the most corresponding gene fragment sequences of mycobacteria deposited in NCBI Nucleotide database. Phylogenetic trees were constructed using the neighbor-joining method (Satou and Nei, 1987) and validated using the maximum composite likelihood method. One thousand bootstrap replicates were run and *Nocardia farcinica* was used as an outgroup.

**Figure 1:**
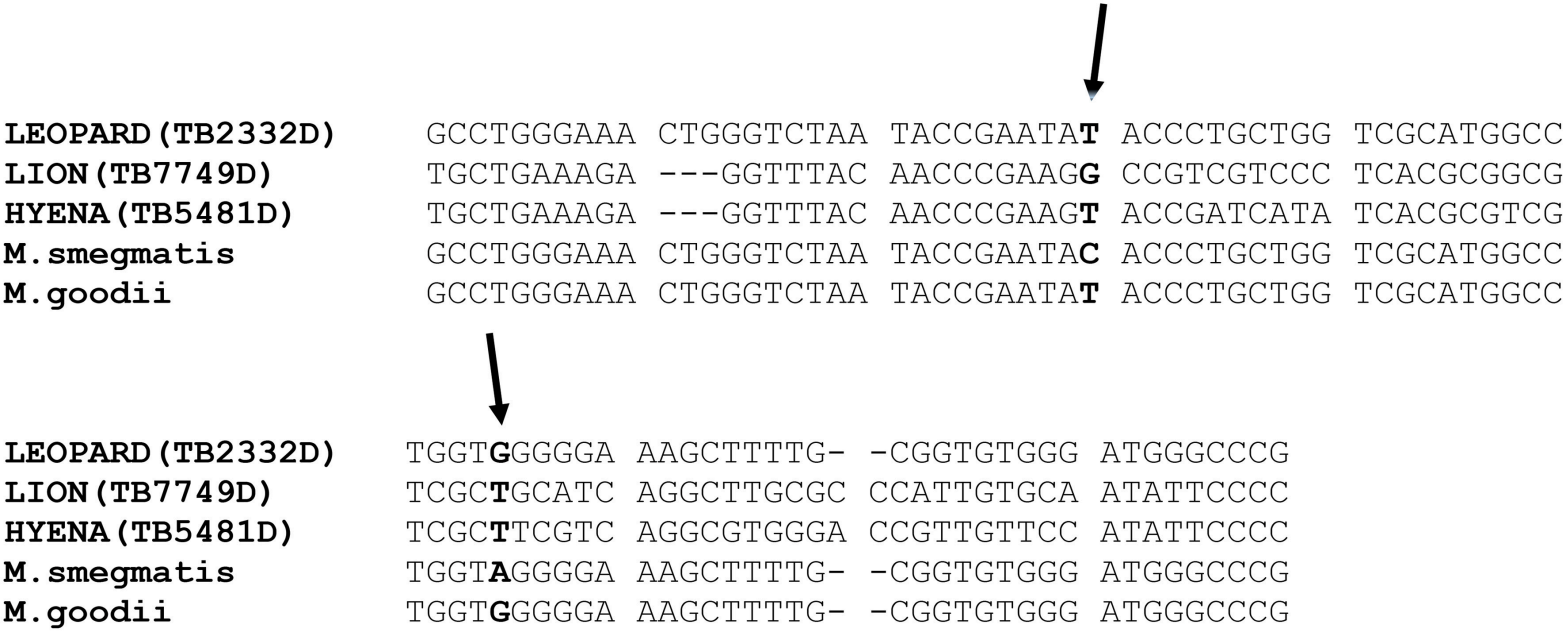
**Alignment of part of the 16S rDNA sequences of three isolates and those of the reference ***M. goodii*** and ***M. smegmatis*** retrieved from NCBI nucleotide database (https://www.ncbi.nlm.nih.gov/nucleotide/) demonstrating similarity between the isolates from leopard to ***M. goodii*** as well as sequence differences between the isolates from hyena and lion to ***M. goodii*** and ***M. smegmatis*****. The 16S rDNA sequence from the lion was submitted to Genbank database and the accession number is KX670576. The two arrows point to single nucleotide differences that exist between *M. smegmatis* and *M. goodii* are in this part of the 16S rDNA.

Since *M. smegmatis* and *M. goodii* have similar 16S rDNA sequences, isolates belonging or closely related to these two species were differentiated by single nucleotide polymorphism (SNP) analysis of their 16S rDNA sequences (Figure 1; van Helden et al., 2008). Alignment of part of the isolate's 16S rDNA as well as that of *M. goodii* and *M. smegmatis* reference sequences extracted from NCBI Nucleotide (https://www.ncbi.nlm.nih.gov/nuccore) database was also performed using MEGA version 7. SNP identification was done manually.

## Results

The diversity of NTM species isolated from different wild animals in South Africa is presented in Table 2. The 16S rDNA and *hsp65* gene sequence analysis revealed that, of 102 isolates analyzed, 96 belong to 30 known Mycobacterium species, two belonged to a species closely related to *Mycobacterium goodii* and *M. smegmatis* and six represented species that could not be identified from the NCBI database (<95% identical to species in NCBI databse). SNP analysis of part of the 16S rDNA of two isolates closely related to *M. goodii* and *M. smegmatis* and *M. smegmatis* ATCC 19420 and *M. goodii* ATCC 700504 reference strains revealed differences between these two isolates and *M. goodii* and *M. smegmatis*, suggesting that these are potentially novel species. The 16S rDNA fragment sequence of one of the isolate closely related *M. goodii/M. smegmatis* originating from a lion sample with tuberculosis-like lesions was submitted to the Genbank database (https://www.ncbi.nlm.nih.gov/genbank) and the accession number is KX670576.

**Table 2:** **NTM species diversity in wildlife and associated pathological changes**.

**NTM species identified (*n*)**	**Host species (*n*)**	**Sample/tissue NTM isolated from**	**Pathological changes observed (positive/total)**
*M. fortuitum*	Meerkat (1)	Tracheal flushing	
	Lion (5)	Head, thoracic, bronchial, peripheral lymph node, bronchial wash	TST positivity (3/5)
			Co-infection of the animal with MTBC (5/5)
			Tuberculosis-like lesions (0/5)
	Mongoose (1)	Testicles	TST positivity (ND)
			Co-infection of the animal with MTBC (0/1)
			Tuberculosis-like lesions (0/1)
	Geneta tigrina (1)	Lung	TST positivity (ND)
			Co-infection of the animal with MTBC (0/1)
			Tuberculosis-like lesions (0/1)
*M. abscessus* subsp. *bolletii*	Lion (33)	Bronchial LN, abdominal LN, mesenteric LN, popliteal LN, thoracic LN, peripheral LN, mammary LN, head LN, lung, axillary LN, bone marrow	TST positivity (ND)
			Co-infection of the animal with MTBC (3/33)
			Tuberculosis-like lesions (1/33)
	Hyena (2)	Head LN, thoracic LN	TST positivity (ND)
			Co-infection of the animal with MTBC (0/1)
			Tuberculosis-like lesions (0/1)
	Leopard (2)	Head LN, peripheral LN	TST positivity (ND)
			Co-infection of the animal with MTBC (0/2)
			Tuberculosis-like lesions (0/2)
*M. gastri* (1)	Nyala (1)	Mesenteric and bronchial LN	TST positivity (ND)
			Co-infection of the animal with MTBC (0/1)
			Tuberculosis-like lesions (1/1)
*M. fluoranthenivorans*	Lion (2)	Head, mesenteric LN	TST positivity (ND)
			Co-infection of the animal with MTBC (2/2)
			Tuberculosis-like lesions (0/2)
*M. acapulcensis/M. flavescence*	Lion (1)	Peripheral LN	TST positivity (ND)
			Co-infection of the animal with MTBC (0/1)
			Tuberculosis-like lesions (0/1)
*M. brasiliensis*	Blesbok (1)	Lung and heart	TST positivity (ND)
			Co-infection of the animal with MTBC (1/1)
			Tuberculosis-like lesions (1/1)
	Mongoose (1)	Testicles	TST positivity (ND)
			Co-infection of the animal with MTBC (0/1)
			Tuberculosis-like lesions (0/1)
	White rhino (1)	Abdominal LN	TST positivity (ND)
			Co-infection of the animal with MTBC (1/1)
			Tuberculosis-like lesions (0/1)
	Lichtenstein hartebeest (1)	Head LN	TST positivity (ND)
			Co-infection of the animal with MTBC (0/1)
			Tuberculosis-like lesions (1/1)
*Mycobacterium* sp. *GR-2007*	Lion (1)	LN	TST positivity (ND)
			Co-infection of the animal with MTBC (0/1)
			Tuberculosis-like lesions (1/1)
*M. elephantis*	Lion (1)	Cranial LN	TST positivity (ND)
			Co-infection of the animal with MTBC (0/1)
			Tuberculosis-like lesions (0/1)
*M. yongonense/intracellulare/M. marseilense*	Mongoose (1)	Lung	TST positivity (ND)
			Co-infection of the animal with MTBC (0/1)
			Tuberculosis-like lesions (0/1)
*M. intracellulare*	Mongoose (1)	Spleen	TST positivity (ND)
			Co-infection of the animal with MTBC (0/1)
			Tuberculosis-like lesions (0/1)
	Wild cat (1)	LN	TST positivity (ND)
			Co-infection of the animal with MTBC (0/1)
			Tuberculosis-like lesions (0/1)
*M. rhodesiae*	Lion (1)	Elbow hygroma	TST positivity (ND)
			Co-infection of the animal with MTBC (1/1)
			Tuberculosis-like lesions (0/1)
*M. simiae*	Cheetah (1)	Peripheral LN	TST positivity (ND)
			Co-infection of the animal with MTBC (0/1)
			Tuberculosis-like lesions (0/1)
	Vervet monkey (1)	Spleen	TST positivity (1/1)
			Co-infection of the animal with MTBC (0/1)
			Tuberculosis-like lesions (0/1)
*M. bouchedurhonense*	Eland (1)	Bronchial LN	TST positivity (ND)
			Co-infection of the animal with MTBC (0/1)
			Tuberculosis-like lesions (1/1)
	Leopard (1)	Head LN	TST positivity (ND)
			Co-infection of the animal with MTBC (0/1)
			Tuberculosis-like lesions (0/1)
*M. duvalii*	Lion (1)	Peripheral LN	TST positivity (ND)
			Co-infection of the animal with MTBC (0/1)
			Tuberculosis-like lesions (0/1)
*M. peregrinum*	Orang-utan (1)	Above ear swab	TST positivity (ND)
			Co-infection of the animal with MTBC (0/1)
			Tuberculosis-like lesions (0/1)
	Lion (1)	Peripheral LN	TST positivity (ND)
			Co-infection of the animal with MTBC (0/1)
			Tuberculosis-like lesions (0/1)
*M. palustre*	Lion (1)	Peripheral and abdominal LN pool	TST positivity (ND)
			Co-infection of the animal with MTBC (0/1)
			Tuberculosis-like lesions (0/1)
	Leopard (1)	Lung and mesenteric LN	TST positivity (ND)
			Co-infection of the animal with MTBC (0/1)
			Tuberculosis-like lesions (0/1)
*M. avium* subsp. *avium*	Lion (2)	Thoracic LN	TST positivity (ND)
			Co-infection of the animal with MTBC (0/2)
			Tuberculosis-like lesions (0/2)
	Eland (1)	Bronchial LN	TST positivity (ND)
			Co-infection of the animal with MTBC (0/1)
			Tuberculosis-like lesions (1/1)
	Hyena (1)	Peripheral LN	TST positivity (ND)
			Co-infection of the animal with MTBC (0/1)
			Tuberculosis-like lesions (0/1)
	African rodent (1)	Lung	TST positivity (ND)
			Co-infection of the animal with MTBC (0/1)
			Tuberculosis-like lesions (0/1)
*M. goodii*	Leopard (1)	Peripheral LN	TST positivity (ND)
			Co-infection of the animal with MTBC (0/1)
			Tuberculosis-like lesions (0/1)
*Mycobacterium* closely related to *M. goodii/M. smegmatis*	Lion (1)	Thoracic LN	TST positivity (ND)
			Co-infection of the animal with MTBC (0/1)
			Tuberculosis-like lesions (1/1)
	Hyena (1)	Peripheral LN	TST positivity (ND)
			Co-infection of the animal with MTBC (1/1)
			Tuberculosis-like lesions (0/1)
*M. virginiense*	Lion (1)	Thoracic LN	TST positivity (ND)
			Co-infection of the animal with MTBC (1/1)
			Tuberculosis-like lesions (0/1)
*M. vulneris*	Mongoose (1)	Spleen	TST positivity (ND)
			Co-infection of the animal with MTBC (0/1)
			Tuberculosis-like lesions (0/1)
	White rhino (1)	Pre-scapcular LN	TST positivity (ND)
			Co-infection of the animal with MTBC (0/1)
			Tuberculosis-like lesions (0/1)
*M. sherrisiii*	Lion (1)	Mesenteric LN	TST positivity (ND).
			Co-infection of the animal with MTBC (0/1)
			Tuberculosis-like lesions (0/1)
*M. hecheshornense DSM 4428/M. sydneyiensis/M. xenopi “Hyn” Wue-TB939/99*	Vervet monkey (1)	Spleen	TST positivity (ND)
			Co-infection of the animal with MTBC (0/1)
			Tuberculosis-like lesions (0/1)
*M. parafortuitum*	Bush buck (1)	Pre-scapcular LN	TST positivity (ND)
			Co-infection of the animal with MTBC (0/1)
			Tuberculosis-like lesions (0/1)
*M. porcinum*	Black wildebeest (1)	Pooled Organs	TST positivity (ND)
			Co-infection of the animal with MTBC (0/1)
			Tuberculosis-like lesions (0/1)
	Lion (3)	Thoracic LN, head LN, ulna attachment	TST positivity (ND)
			Co-infection of the animal with MTBC (0/3)
			Tuberculosis-like lesions (0/3)
*M. vaccae/M. vanbaalenii*	Lion (1)	Head LN	TST positivity (ND)
			Co-infection of the animal with MTBC (1/1)
			Tuberculosis-like lesions (0/1)
*M*. sp. *NJB901/M*.sp. *B6. 10-23/M*. sp. *NLA001000256/M*. sp. *CST-8.2b/M. chelonae/M. fuerth*	Leopard (1)	Lung	TST positivity (ND)
			Co-infection of the animal with MTBC (1/1)
			Tuberculosis-like lesions (0/1)
*Mycobacterium* sp. WCM 7299	Mongoose (1)	Liver	TST positivity (ND)
			Co-infection of the animal with MTBC (0/1)
			Tuberculosis-like lesions (0/1)
*Mycobacterium* sp. GPK 1020	Mongoose (1)	Nose	TST positivity (ND)
			Co-infection of the animal with MTBC (0/1)
			Tuberculosis-like lesions (0/1)
*M. sinense* JMD601	Lion (4)	Head LN, thoracic LN, retropharyngeal LN	TST positivity (ND)
			Co-infection of the animal with MTBC (0/4)
			Tuberculosis-like lesions (1/4)
*M. septicum/M. peregrinum*	Lion (1)	Organ	TST positivity (ND)
			Co-infection of the animal with MTBC (0/1)
			Tuberculosis-like lesions (1/1)
	Black wildebeest (1)	Peripheral LN	TST positivity (ND)
			Co-infection of the animal with MTBC (0/1)
			Tuberculosis-like lesions (1/1)
Unidentified Mycobacterial species	Lion (5)	Lung, liver, head LN, popliteal LN, bronchial wash	TST positivity (ND)
			Co-infection of the animal with MTBC (0/5)
			Tuberculosis-like lesions (0/5)
	Kudu (1)	LN	TST positivity (ND)
			Co-infection of the animal with MTBC (0/1)
			Tuberculosis-like lesions (0/1)

*MTBC, Mycobacterium tuberculosis complex; ND, Not determined*.

Of the 102 isolates analyzed, 37 (36.3%) were identified as *Mycobacterium abscessus* subsp. *bolletii* by sequencing of both 16S rDNA and the *hsp65* gene fragments. Thirty-three of the 37 isolates originated from lion samples, two from hyenas and another two from leopards. The second most frequently isolated species was *M. fortuitum* (*n* = 8) isolated from lions (*n* = 5), meerkat (*n* = 1), geneta tigrina (*n* = 1), and mongoose (*n* = 1); followed by *M. sinense* JMD601 (*n* = 4) with all isolates originating from lion samples; *M. brasiliensis* (*n* = 4) isolated from blesbok (*n* = 1), mongoose (*n* = 1), white rhino (*n* = 1) and Lichtenstein hartebeest (*n* = 1); and finally *M. porcinum* (*n* = 4) isolated from lions (*n* = 3) and a black wildebeest (*n* = 1). The other NTM species were identified in low numbers of <4 and these include: *M. goodii, M. gastri, M. fluoranthenivorans, M. acapulcensis/M. flavescence, Mycobacterium* sp. GR-2007, *M. elephantis, M. intracellulare, M. yongonense/intracellulare/M. marseilense, M. rhodesiae, M. simiae, M. bouchedurhonense, M. duvalii, M. peregrinum, M. palustre, M. avium* subsp. *avium, M. virginiense, M. sherrisiii, M. vulneris, M. hecheshornense DSM 4428/M. sydneyiensis/M. xenopi “Hyn” Wue-TB939/99, M. parafortuitum, M*. sp. *NJB901/M.sp. B6. 10-23/M*. sp. *NLA001000256/M*. sp. *CST-8.2b/M. chelonae/M. fuerth, M. vaccae/M. vanbaalenii, Mycobacterium* sp. WCM 7299, *Mycobacterium* sp. GPK 1020, *M. septicum/M. peregrinum*, and *Mycobacterium* species closely related to *M. goodii/M. smegmatis*.

**Figure 2:**
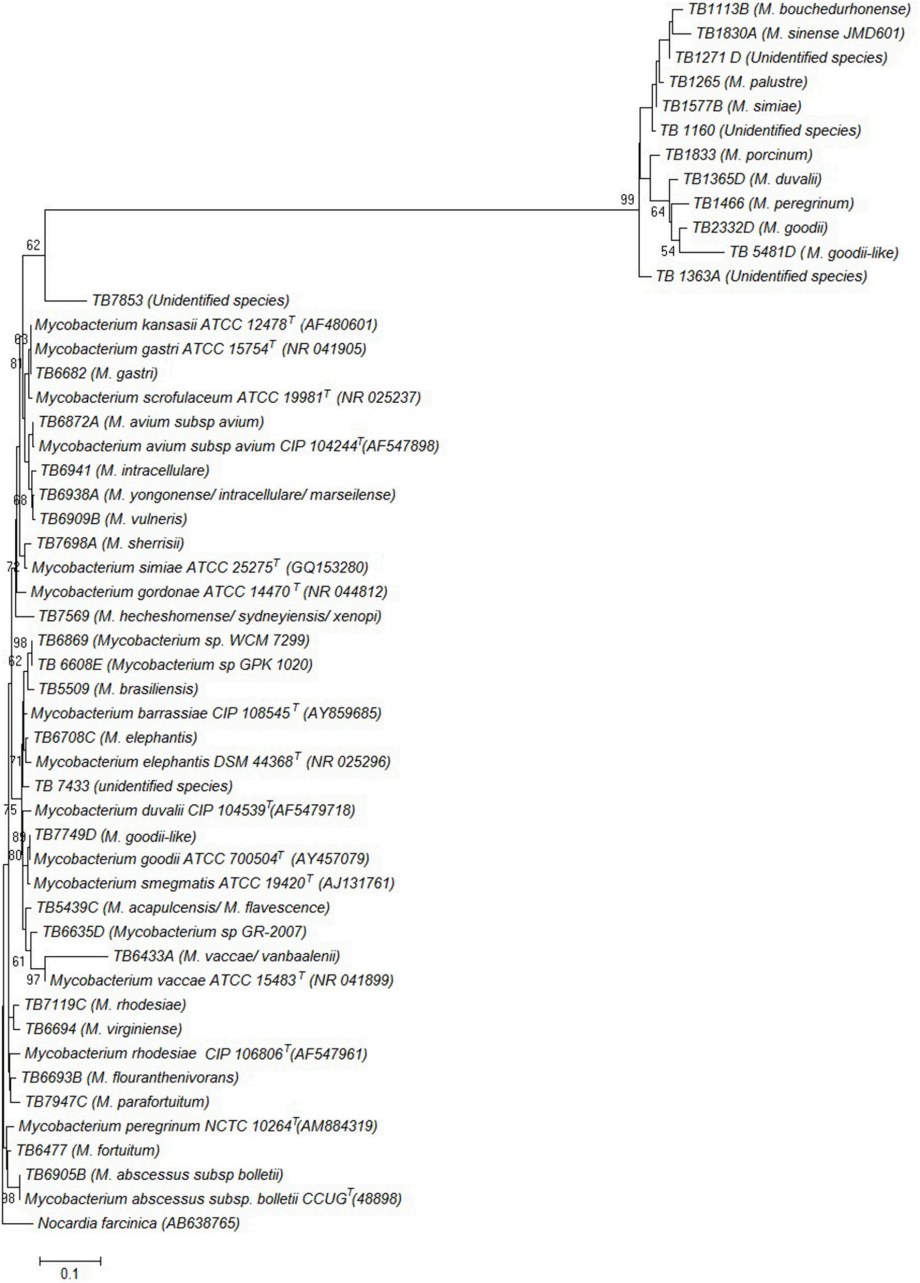
**Phylogenetic tree constructed using neighbor joining method, illustrating the genetic position of the isolates**. The species that the isolates were found to belong to are shown in parenthesis next to each isolate. Genbank accession numbers for the reference sequences are also shown in parenthesis. The tree is based on the partial 16S rDNA gene sequences. The percentage of replicate trees in which the associated taxa clustered together in the bootstrap test (1000 replicates) are shown next to the branches. *Nocardia farcinica* was used as an out group sequence. Evolutionary analyses were conducted in MEGA7.

Phylogenetic relatedness of the isolates representative of each species identified in this study, and other *Mycobacterium* species, based on the 16S rDNA sequences is illustrated by the phylogenetic tree in Figure 2.

To investigate if the NTM species isolated could have potentially caused tuberculous-like or cross-reactive immune responses, records relating to pathological changes including the formation of tuberculosis-like lesions and the tuberculin skin test positivity of the animals were analyzed. Information about whether more than one Mycobacterium species including *Mycobacterium tuberculosis* complex (MTBC) or another NTM were isolated from a single sample showing tuberculosis-like lesion was analyzed in-order to eliminate the possibility or any doubt or confusion about which mycobacterial species could have potentially caused lesions. However, we did not investigate other pathogens other than Mycobacteria as potential causes of lesions, a move that could have strengthened findings of certain NTM species identified in this study, as opportunistic pathogens of animals. A similar analysis was done at animal level (Investigating if isolation of more than one Mycobacterium species from the same animal). Likewise, information about isolation of more than one Mycobacterium species from the TST positive reactors was also investigated for elimination of any doubt about which mycobacteria could have elicited an immune response. The summary of the data is presented in Table 2.

Tuberculosis-like lesions were observed in the mesenteric and bronchial lymph nodes of nyala; lung, heart and head lymph node of blesbok, and haartebeest; bronchial lymph nodes of an eland and a lion; retropharyngeal lymph nodes and lung of lion. In all these cases, pure cultures of the following NTM species or subspecies were isolated and no any other mycobacterial species was isolated from the same animal or sample: *M. abscessus* subsp. *bolletii* isolated from lion, *Mycobacterium gastri* was isolated from nyala; *Mycobacterium brasiliensis* from blesbok and hartebeest; *Mycobacterium avium* subsp. *avium* from eland; *Mycobacterium* sp. GR-2007, *Mycobacterium sinense* JMD601, *Mycobacterium* species closely related *Mycobacterium goodii*/*Mycobacterium smegmatis*, and *Mycobacterium septicum*/*M. peregrinum* were all isolated from lions. Co-infection of the host animal species with *M. bovis/M. tuberculosis* and the following NTM species was observed: *M. abscessus* subspecies *bolletii, M. fluoranthenivorans, M. brasiliensis, M. rhodesiae, Mycobacterium* species closely related to *M. goodii/M. smegmatis, M. virginiense, M. vaccae/M. vanbaalenii* and finally, *M*. sp. *NJB901/M*.sp. *B6. 10-23/M*. sp. *NLA001000256/M*. sp. *CST-8.2b/M. chelonae/M. fuerth* in different animal species as shown in Table 2.

A pure culture of *Mycobacterium simiae* was isolated from TST positive reactor vervet monkey. No other mycobacterial species was isolated from this animal. In other cases, where NTM were isolated, there were either no visible lesions or no information regarding TST (Table 2).

## Discussion

In this study 30 known NTM species, a species closely related to *Mycobacterium goodii* and *M. smegmatis* (comprising of two isolates) as well-unidentified species (comprising of six isolates) were detected from 102 isolates originating from wild animals in South Africa and Botswana, collected between the years 1998 and 2010. These findings demonstrate a wide diversity of NTM species circulating in the wildlife ecosystems of Southern Africa. Furthermore, isolation of strains belonging to unidentified species suggests the occurrence of a number of uncharacterized potentially novel NTM species in wildlife. In this study except for a species found to be closely related to *M. goodii* and *M. smegmatis*, they were not associated with any pathological changes in their hosts and as such, their roles in causing diseases and in eliciting an immune response are not fully known. With the use of a combination of molecular assays viz sequencing of the 16S rDNA and the *hsp65* genes as well as SNP analysis of the 16S rDNA, screening of the isolates for *IS900* and *Mycobacterium avium* specific 16S rDNA gene fragments, we were able to delineate closely related species as well as identification of some NTM to subspecies level. The NTM identified in this study comprise species of which data for their clinical and veterinary significance have been reported previously (known pathogens), which have been previously described as opportunistic pathogens; as well as species that have not been previously reported to cause any diseases. The isolation of these NTM groups (known pathogens as well as those whose pathogenicity has not been reported in animals) from tissue samples with tuberculosis-like lesions in this study suggests their potential role as sources of infection in those particular animals. These include *Mycobacterium abscessus* subsp. *bolletii, Mycobacterium gastri, Mycobacterium* closely related to *Mycobacterium goodii, Mycobacterium brasiliensis, Mycobacterium sinense* JMD 601, *Mycobacterium avium* subsp. *avium, Mycobacterium* sp. GR-2007, *Mycobacterium septicum*/*M. peregrinum*, and *Mycobacterium bouchedurhonense. Mycobacterium abscessus* subspecies *bolletii* together with *Mycobacterium abscessus* subspecies *abscessus* and *Mycobacterium abscessus* subspecies *massiliense* constitute the *Mycobacterium abscessus* group. These rapidly growing Mycobacteria (RGM) have been identified not only as sources of pulmonary infections but as sources of nosocomial, skin and soft tissue infections, osteoarticular infections as well as disseminated diseases in humans (Viana-Niero et al., 2008; Piersimoni and Scarparo, 2009; Zelazyn et al., 2009; Colombo et al., 2012). Another significance of these NTM is their high resistance to antibiotics (Nessar et al., 2012). The different subspecies however differ from each other in their antibiotic resistance profile (Nessar et al., 2012). Information on the pathogenic role of *Mycobacterium abscessus* subspecies *bolletii* in animals is still scarce. This is the first study to report *M. abscessus* subspecies *bolletii* as a probable cause of lesions in a peripheral lymph node of a lion in South Africa. *Mycobacterium abcessus* has been isolated in various fish species and has been described to cause diseases in humans (Bercovier and Vincent, 2001). In a study in Asia, *M. abscessus* was found to be the second most clinically relevant NTM, after *Mycobacterium avium* subsp. *avium*, responsible for pulmonary diseases (Simons et al., 2011). *M. abscessus* has also been previously reported in animals of South Africa, including wildlife, but not necessarily as a cause of diseases (Botha et al., 2013; Gcebe et al., 2013). In three other cases in this study, co-infection with *M. bovis* and this NTM was observed in lions. Isolation of *M. abscessus* subspecies *bolletii* together with *M. bovis* from sample presenting tuberculosis-like lesions could indicate the role of this NTM as an opportunistic pathogen. Contrary, to what has been reported in wildlife NTM diversity studies in Ethiopia, Tanzania, and buffaloes in South Africa, *M. abscessus* subsp. *bolletii* was isolated most frequently in this study mainly from lions (Tschopp et al., 2010; Gcebe et al., 2013; Katale et al., 2014).

Only a few reports of *Mycobacterium gastri* as a cause of disseminated and pulmonary mycobacteriosis diseases in humans have been published (Velayati et al., 2005; Guerra et al., 2008). Our study presents a first report of *Mycobacterium gastri* isolation in South Africa from an animal species (nyala) which presented tuberculosis -like lesions. Investigation of the pathogenic role of this NTM in animals is therefore necessary.

*Mycobacterium avium* subspecies *avium* is a well-known and mostly studied animal pathogen (Rastogi et al., 2001). We isolated this species/subspecies from a bronchial lymph node of an eland with lesions, confirming its status as an animal pathogen. It was also isolated from four other animal samples: hyena, African rodent, and two lions without any visible lesions.

*Mycobacterium sinense* JMD601 is a recently described pathogenic NTM, first isolated from a human patient with tuberculosis- like disease (Zhang et al., 2013). Rónai et al. (2016) have reported isolation of this species from wild boar, fallow deer, swine, and cattle from Hungary. We isolated this pathogenic NTM from a lion which also showed tuberculosis-like lesions, as well as three other lion samples with no apparent lesions.

*Mycobacterium brasiliensis* has been isolated from humans before, but its clinical relevance is still largely unknown (Chin'ombe et al., 2016). Reports of isolation of this species from animals are scarce. In this study we isolated this species from a Lichtenstein hartebeest head lymph node without MTBC, presenting tuberculosis like-lesions, suggesting its pathogenic potential in this animal species. This NTM was isolated in this study from samples from three other animals namely, blesbok, mongoose, and white rhino, showing no visible lesions. Botha et al. (2013) have reported isolation of this NTM from wildlife of South Africa. It is important to examination this NTM's role in disease causation in animals.

*Mycobacterium bouchedurhonense* was isolated in this study from an eland bronchial lymph node, representing tuberculosis-like lesions. This recently described member of MAC is being reported for the first time in South African wildlife.

We have also isolated *Mycobacterium* sp. GR-2007 from a lymph node of a lion with lesions. This is not a validly published Mycobacterium species, and there are no reports of its occurrence. However, its isolation from a lymph node with a tuberculosis-like lesion may imply its pathogenic nature and the need for further characterization of this NTM.

*Mycobacterium goodii* is a RGM species closely related to *M. smegmatis. Mycobacterium goodii* was reported to have caused infection in a hyena in South Africa as well as in African rodents in Tanzania (van Helden et al., 2009; Durnez et al., 2011). We identified a Mycobacterium species closely related to *M. goodii* and *M. smegmatis* from a lion lymph node with tuberculosis-like lesions. This NTM is not in the public databases and as such it could be a potential novel NTM species and therefore needs to be further characterized with a particular focus on its pathogenicity.

It has also been hypothesized that mycobacterial infections do not necessarily need to be established and maintained by animal species, colonization without casing disease may be enough for development of immune responses. Some NTM species, notably *Mycobacterium kansasii, Mycobacterium marinum*, and *M. fortuitum* have been reported to induce anti-mycobacterial immune responses that interfere with the diagnosis of bovine tuberculosis either by TST or gamma interferon tests (Vordermeier et al., 2007; Michel et al., 2011). *Mycobacterium simiae* was isolated in this study from as sample originating from a tuberculin skin test positive monkey, demonstrating its potential to elicit inappropriate immune responses in monkeys that may interfere with diagnosis of tuberculosis by immunological tests (Schiller et al., 2010). It may therefore be important to investigate the role of this NTM in eliciting cross-reactive immune responses against *Mycobacterium bovis* antigens. *M. simiae* has been reported to cause diseases in monkeys not presenting any specific pathology and has also been isolated from fish (Bercovier and Vincent, 2001). This species has previously been reported in cattle, African buffaloes, and the environments in South Africa (Gcebe et al., 2013). It has also been isolated from cattle and humans in Tanzania and from the environment in Uganda (Kankya et al., 2011; Katale et al., 2014).

We also identified other species that have been shown to be of clinical significance in human mycobacterial diseases in several studies, which in this study were isolated from specimen without lesions. These notably include members of the *Mycobacterium avium* complex: *M. yongonense/intracellulare/M. marseilense, M. intracellulare* as well as *M. sherrisii, Mycobacterium porcinum*, and *M. virginiense. Mycobacterium sherrisii* is a slow growing emerging human Mycobacterial pathogen, particularly isolated from HIV positive patients who may develop invasive and disseminated mycobacteriosis diseases (van Ingen et al., 2011). Botha et al. (2013) had reported isolation of this Mycobacterium species from wild animals in South Africa (Botha et al., 2013). *Mycobacterium virginiense* is a recently described member of the *M. terrae* complex reported to cause major tenosynovitis/osteomyelitis in humans (Vasireddy et al., 2016). Here, this species was isolated from a lion co-infected with *M. bovis* and its possible role as an opportunistic pathogen of animals need to be investigated. We report isolation of this Mycobacterium species for the first time in animals in South Africa. *Mycobacterium porcinum* was first isolated from porcine lymph nodes presenting tuberculosis-like lymphadenitis (Tsukamura et al., 1983). In this study, this species was isolated from lions and black wildebeest. The relevance of the MAC species, viz *M. yongonense/M. marseilense/M. intracellulare* in disease causation or possible cross-reactivity with *M. bovis* antigens was not established in our case. *Mycobacterium yongonense* is a recently described member of MAC isolated from patients with pulmonary symptoms (Kim et al., 2013) while *M. intracellulare* is a known pathogen of humans (van Ingen et al., 2009). This MAC species has been isolated from cattle in Tanzania, and Uganda as well as from wildlife in South Africa (Kankya et al., 2011; Botha et al., 2013; Katale et al., 2014). Other NTM species isolated in this study from specimen not presenting tuberculosis-like lesions include *M. fortuitum, M. fluoranthenivorans, M. elephantis, M. vulneris, M. rhodesiae, M. duvalii, M. peregrinum, M. palustre, M. vaccae/M. vanbaalenii, M. parafortuitum, Mycobacterium* sp. WCM 7299, and *Mycobacterium* sp. GPK 1020. These NTM have been isolated from animals as well the environment in several studies (Bercovier and Vincent, 2001; Botha et al., 2013; Gcebe et al., 2013, 2016; Katale et al., 2014). *Mycobacterium fortuitum* and *M. vulneris* have both been found to be associated with pulmonary infections in humans (Simons et al., 2011; Hoefsloot et al., 2013). The role of these mycobacterial species as causative agents of disease as well as their possible influence in bovine tuberculosis diagnostics has not been established in this study.

In conclusion, we have elucidated the diversity of NTM species circulating in different wild animals in South Africa. Thirty known NTM species/subspecies as well as unidentified NTM, were detected in this study, of which nine were isolated from animal specimen showing tuberculosis-like lesions, highlighting the importance of these NTM as potential pathogens of animals. In addition, isolation of unidentified NTM species, suggests the occurrence of a number of uncharacterized potentially novel NTM species in wildlife, whose roles are unknown. Isolation of *Mycobacterium simiae* from a positive TST reactor vervet monkey free of MTBC demonstrate this NTM species' ability to induce cross-reactive immune responses against MTBC antigens. *Mycobacterium* abscesses subsp. *bolletti* was found to be the most frequently occurring NTM, isolated mainly from lions. Knowledge gained in this study contribute to the understanding of NTM species circulating in wild animals in South Africa and the pathogenic potential of certain species as well as to a certain extent their potential to hamper bTB immunological diagnostic assays.

## Author contributions

All authors listed, have made substantial, direct and intellectual contribution to the work, and approved it for publication.

### Conflict of interest statement

The authors declare that the research was conducted in the absence of any commercial or financial relationships that could be construed as a potential conflict of interest.
